# Fatal Asphyxiation in Two Long-Finned Pilot Whales (*Globicephala melas*) Caused by Common Soles (*Solea solea*)

**DOI:** 10.1371/journal.pone.0141951

**Published:** 2015-11-18

**Authors:** Lonneke L. IJsseldijk, Mardik F. Leopold, Elisa L. Bravo Rebolledo, Rob Deaville, Jan Haelters, Jooske IJzer, Paul D. Jepson, Andrea Gröne

**Affiliations:** 1 Department of Pathobiology, Faculty of Veterinary Medicine, Utrecht University, Utrecht, The Netherlands; 2 Department of Ecosystems, Wageningen IMARES, Den Burg, Texel, The Netherlands; 3 UK Cetacean Strandings Investigation Programme, Institute of Zoology, Regent’s Park, London, United Kingdom; 4 Royal Belgian Institute of Natural Sciences (RBINS), Operational Directorate Natural Environment, Oostende, Belgium; New York Institute of Technology College of Osteopathic Medicine, UNITED STATES

## Abstract

Long-finned pilot whales (*Globicephala melas*) are rare visitors to the southern North Sea, but recently two individual strandings occurred on the Dutch coast. Both animals shared the same, unusual cause of death: asphyxiation from a common sole (*Solea solea*) stuck in their nasal cavity. This is a rare cause of death in cetaceans. Whilst asphyxiation has been reported in smaller odontocetes, there are no recent records of this occurring in *Globicephala* species. Here we report the stranding, necropsy and diet study results as well as discuss the unusual nature of this phenomenon. Flatfish are not a primary prey species for pilot whales and are rarely eaten by other cetaceans, such as harbour porpoises (*Phocoena phocoena*), in which there are several reports of asphyxiation due to airway obstruction by soles. This risk may be due to the fish’s flexible bodies which can enter small cavities either actively in an attempt to escape or passively due to the whale ‘coughing’ or ‘sneezing’ to rid itself of the blockage of the trachea. It is also possible that the fish enter the airways whilst the whale is re-articulating the larynx after trying to ingest large, oddly shaped prey. It is unlikely that the soles entered the airways after the death of the whales and we believe therefore that they are responsible for the death of these animals.

## Introduction

Long-finned pilot whales (*Globicephala melas*) occur in temperate and sub-polar waters, including both oceanic and coastal waters of the North Atlantic [1,2], where surveys yielded an abundance estimate of 778,000 individuals (coefficient of variation = 0.30) [3]. The long-finned pilot whale has no species conservation status as knowledge of population boundaries is still incomplete [2,4].

In the North Atlantic, long-finned pilot whales occur mostly in deep offshore waters [2,3] and along the edges of continental shelves [5]. Pilot whale occurrence is believed to be prey-driven [2,6]. Their diet is dominated by cephalopods, but can also comprise a variety of fish species, including both pelagic and demersal roundfish, flatfish, and several species of invertebrates other than squid [7–14].

In the North Sea, long-finned pilot whales mainly occur at its fringes, both in the north, from the Norwegian Deep to the Faroe Islands and in the south, from the Bay of Biscay to the English Channel [5]. The species is a rare visitor to the southern North Sea with, for example, only 17 strandings in The Netherlands since 1581 (www.walvisstrandingen.nl), [15] and only two strandings on the English coast in the southern North Sea region (UK strandings database, 1990–2014). However, recently two individual strandings occurred on the Dutch coast with both animals remarkably sharing an unusual cause of death. Here we describe these two rare strandings in detail, including the post-mortem findings and diet analyses, adding to what is known about the long-finned pilot whale’s life history, feeding ecology and pathology.

## Methods

The two animals described in this study were not used for scientific or commercial testing. Both were free-living whales which died in their natural environment and were washed ashore in The Netherlands. No consent from an Animal Use Committee is required in The Netherlands when using tissues from animals that died due to natural causes, as was the case in these two long-finned pilot whales. Consequently, animal ethics committee approval was not applicable to this work.

### Case histories

A male long-finned pilot whale in a moderate state of decomposition was found at Nieuw-Haamstede (Zeeland, SW Netherlands: 51°42’58”N, 3°41’06”E) on the 17^th^ of December 2014. The entire epidermis was sloughed, all extremities had lost structure and some teeth were missing, but in general the carcass was intact and all changes were interpreted as post-mortem artefacts. It was transported to the Faculty of Veterinary Medicine of Utrecht University the same day, where the necropsy took place the following morning. The whale had a total length of 3.85 m and weighed 410 kg.

A female long-finned pilot whale in an advanced state of decomposition was found near Petten (Noord-Holland, Dutch mainland coast, 52°44’24”N, 4°38’22”E) on the 11^th^ of January 2015. The entire epidermis was sloughed, the dorsal fin and fluke were absent, the pectoral fins had lost structure, all teeth were missing and the right side of the mandible was broken; all changes were interpreted as post-mortem artefacts. The carcass was transported the following morning to Utrecht where the necropsy took place later that day. The whale was estimated to have measured 4–4.5 m and to have weighed over 600 kg.

### Pathological examination

The necropsies were carried out according to a standardized protocol [16]. All organs were examined macroscopically. A standard set of organ samples was collected for histopathological examination. Also, the entire gastrointestinal tracts (GIT) were collected and stored at -20°C prior to the diet study. The occurrence of macro-parasites in all major organs (except for the skin) was investigated, scored for severity (mild, moderate or severe infestation), and parasites were collected in alcohol and identified morphologically by a veterinary parasitologist.

### Additional tests

Both animals were tested for *Brucella ceti* infection: two swabs of the brain of each animal, stored at -20°C prior to analysis, and lung tissue of the female long-finned pilot whale, stored at -80°C prior to analysis, were tested. Culture was performed by the Central Veterinary Institute (CVI; Lelystad, The Netherlands) (described by [17]). The brain swabs were also investigated at the CVI by real-time PCR for the presence of avian influenza virus (following [18]). From the female long-finned pilot whale, two swabs of the aboral end of the intestinal tract contents were submitted to the CVI for investigating the presence of extended spectrum beta-lactamase (ESBL) producing *Escherichia coli* (as described by [19]).

### Diet analysis

The contents of the entire GIT were studied (following methods outlined in [20]). In brief: intact prey and hard parts from partially digested prey were collected from the oesophagus, the nasal cavity, the stomach and intestines, identified to species level, the size of otoliths (corrected for wear) was established, and they were paired where possible. The minimum number of individual prey and the size and mass for each prey was determined. One intact fish was measured directly; all other prey sizes were estimated from various prey hard parts. Fish lengths and masses were estimated from sagittal otoliths (using [21]) or from other hard parts using the reference collection available at Wageningen IMARES. For the squid (*Sepia officinalis*), regression equations were derived linking shell width (SW in mm) to mantle length (L in cm) and body mass (M in grams, excluding the shell), from 18 fresh specimens caught in Dutch waters in December 2014 (L = 0.392*SW-1.5079, R^2^ = 0.95; M = 0.0016*SW^3.3507^, R^2^ = 0.96). The tails of brown shrimp (*Crangon crangon*) were used to estimate body length (cf. [22]).

## Results

### Necropsy findings

Based on total body length and the size of its testes (approximately 12 x 6 cm), the male long-finned pilot whale was a juvenile [23]. Even though its blubber thickness could not be measured due to autolysis, the animal was likely in a normal to good body condition, given its well-developed musculature. The main necropsy finding was the presence of a flatfish, identified as a common sole (*Solea solea*) of 34.6 cm total length, lodged in the nasal cavity. The head of the sole pointed toward the blowhole and 5 cm of the tail was in the oesophagus. The larynx of the whale was displaced to the left. The fish was complete and only mildly decomposed. Stomach contents (partly-digested prey remains) were present in the oesophagus of the whale, suggesting that it had also vomited prior to death. Partly digested prey remains were also present in the fore-stomach (Table 1). Other findings were a moderate parasite infestation of the fore- and second- stomach walls (*Pholeter gastrophilus* (Trematoda, Heterophiidae)) and a mild infestation in the intestine (*Bolbosoma capitatum* (Acanthocephala)). No other significant macroscopic lesions were found.

**Table 1:** Prey species from the gastrointestinal tracts of the two long-finned pilot whales. Prey found in the gastrointestinal tracts of the two long-finned pilot whales (PW-01 and PW-02), with reconstructed total prey lengths and fresh masses from prey hard parts, as indicated.

Idcode	Preynr	Prey species	Scientific name	ID-item measured	N	TPL(cm)	Prey Mass (g)	Remark
PW-01	1	Common sole	*Solea solea*	otoliths	2	34.6	365.3	otoliths taken from the fish's head; sole stuck in nasal cavity
PW-01	2	Sand goby	*Pomatoschistus minutus*	R-otolith	1	5.3	1.3	secondary prey?
PW-01	3	Plaice	*Pleuronectes platessa*	otoliths	2	25.7	186.5	premaxilla also found
PW-01	4	Plaice	*Pleuronectes platessa*	otoliths	2	26.1	197	premaxilla also found
PW-01	5	Herring	*Clupea harengus*	L-otolith	1	19.2	47.5	
PW-01	6	Common cuttlefish	*Sepia officinalis*	cuttlebone	1	8.6	79.0	beaks were also found; body mass without cuttlebone
PW-01	7	Common cuttlefish	*Sepia officinalis*	cuttlebone	1	8.4	75.1	beaks were also found; body mass without cuttlebone
PW-01	8	Common cuttlefish	*Sepia officinalis*	cuttlebone	1	8.6	79.7	beaks were also found; body mass without cuttlebone
PW-01	9	Common cuttlefish	*Sepia officinalis*	cuttlebone	1	7.7	59.8	beaks were also found; body mass without cuttlebone
PW-01	10	Common cuttlefish	*Sepia officinalis*	cuttlebone	1	6.8	43.2	beaks were also found; body mass without cuttlebone
PW-01	11	Common cuttlefish	*Sepia officinalis*	cuttlebone	1	9.6	103.2	beaks were also found; body mass without cuttlebone
PW-01	12	Common cuttlefish	*Sepia officinalis*	cuttlebone	1	6.0	31.2	beaks were also found; body mass without cuttlebone
PW-01	13	Common cuttlefish	*Sepia officinalis*	cuttlebone	1	5.5	25.9	beaks were also found; body mass without cuttlebone
PW-01	14	Common cuttlefish	*Sepia officinalis*	cuttlebone	1	5.4	24.7	beaks were also found; body mass without cuttlebone
PW-01	15	Common cuttlefish	*Sepia officinalis*	cuttlebone	1	5.9	30.8	beaks were also found; body mass without cuttlebone
PW-01	16	Common cuttlefish	*Sepia officinalis*	cuttlebone	1	4.8	18.5	beaks were also found; body mass without cuttlebone
PW-01	17	Common cuttlefish	*Sepia officinalis*	cuttlebone	1	4.3	13.4	beaks were also found; body mass without cuttlebone
PW-01	18	Common cuttlefish	*Sepia officinalis*	cuttlebone	1	3.2	6.7	beaks were also found; body mass without cuttlebone
PW-01	19	Brown shrimp	*Crangon crangon*	tail	1	5.9	2.4	specimen nearly intact—secondary prey?
PW-01	20	Brown shrimp	*Crangon crangon*	tail	1	5.8	2.3	specimen nearly intact—secondary prey?
PW-01	23	Brown shrimp	*Crangon crangon*	tail	1	5.6	2.1	specimen nearly intact—secondary prey?
PW-01	22	Brown shrimp	*Crangon crangon*	tail	0.5	5.0	1.4	identified from loose tail flaps—secondary prey?
PW-01	21	Brown shrimp	*Crangon crangon*	tail	0.5	4.2	0.8	identified from loose tail flaps—secondary prey?
PW-01	24	River lampern	*Lampetra fluviatilis*	upper and lower teeth	1	31	48	only one reference specimen available, size estimated crudely
PW-01	25	River lampern	*Lampetra fluviatilis*	lower teeth	1	31	48	second row of lower teeth, same size as first set
PW-02	1	Common Sole	*Solea solea*	otoliths	1	27.5	181.9	otoliths taken from the fish's head; sole stuck in nasal cavity
PW-02	2	Common Sole	*Solea solea*	whole fish	2	23.0	105.1	Intact fish in oesophagus, measured directly
PW-02	3	European squid	*Loligo vulgaris*	lower & upper beak	1	18.3	123.5	TPL = mantle length
PW-02	4	European squid	*Loligo vulgaris*	lower beak	1	16.8	83.1	TPL = mantle length

**Fig 1:**
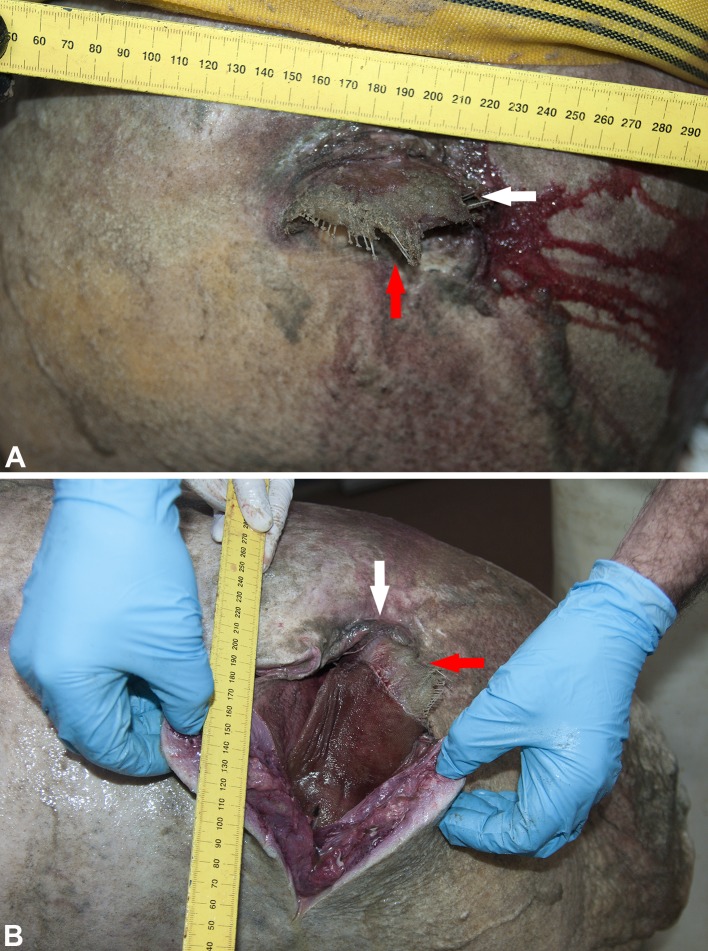
Adult female pilot whale with common sole stuck in nasal cavity. A: external view of sole’s tail hanging out of blowhole. B: blowhole cut open to the right side, revealing the sole in the vestibular sac. The white arrow indicates the left side of the blowhole opening, while the red arrow indicates the fish’s tail in both images.

**Fig 2:**
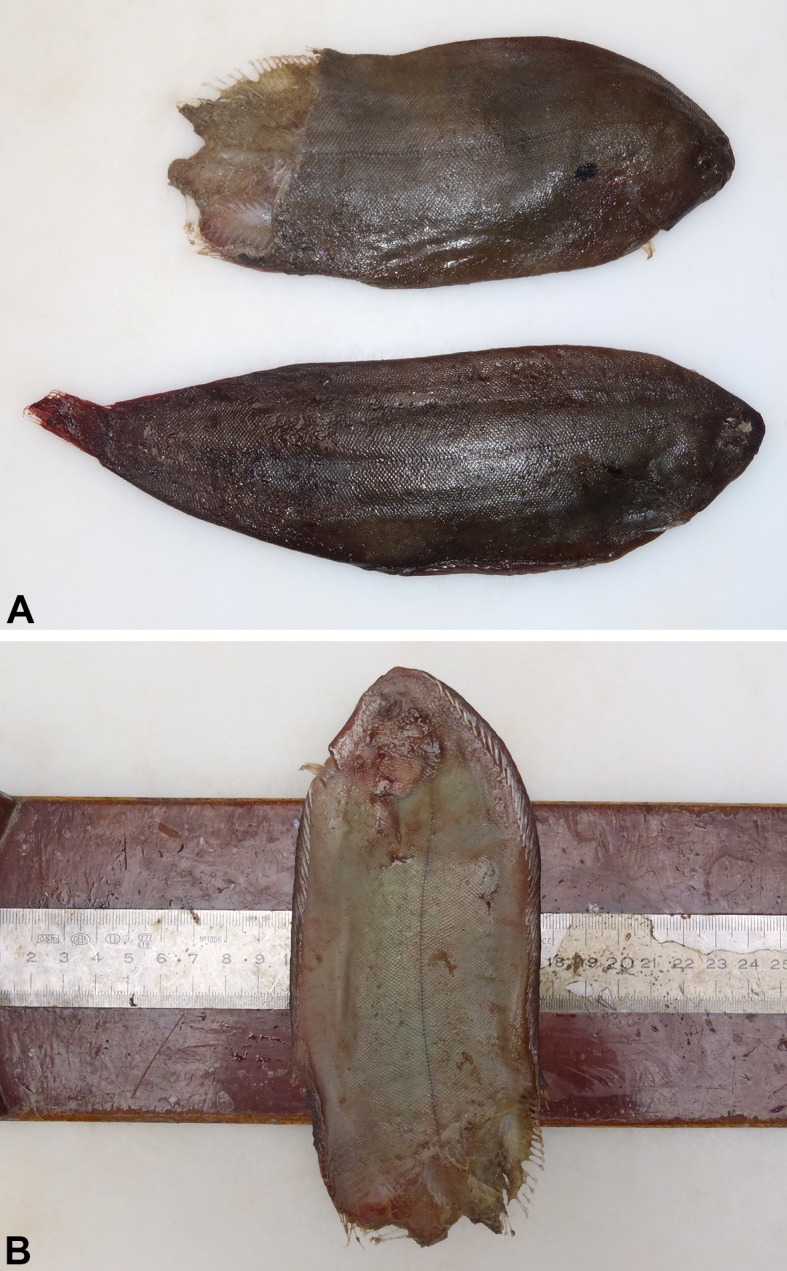
Common soles retrieved from female pilot whale. A: the sole from the nasal cavity (top) and from the oesophagus (down). B: the sole from the nasal cavity showing its body width (7.7 cm).

Based on estimated total length [23] and the state of the ovaries, the female long-finned pilot whale was an adult. The animal was neither lactating nor pregnant, but the ovaries showed *corpora albicantia* revealing earlier pregnancies. It was not possible to determine body condition due to the advanced state of decomposition. The main necropsy finding was the presence of a common sole in the nasal cavity. Unlike the previously described case, approximately 3 cm of the tail of the sole was protruding from the blowhole, while the rest of the body was inside the nasal cavity, with the head pointing downward towards the cavity of the mouth (Fig 1A and 1B). Fish length, estimated from the otolith measurements, was 27.5 cm. Another, slightly smaller sole (23 cm long, measured directly) was found in the oesophagus, laterally to the pharynx. Both soles were intact and only mildly decomposed, except for the section protruding from the blowhole, which was in an advanced state of decomposition (Fig 2A and 2B). Three squid beaks were present in the fore-stomach, while no other prey remains were found in the GIT (Table 1). Other findings were mild parasite infestations of the fore- and second- stomach walls (*Pholeter gastrophilus*) and a mild infestation in the intestine (*Pholeter gastrophilus* and *Bolbosoma capitatum*). No other significant macroscopic lesions were found.

### Histopathological results

The state of decomposition of both carcasses hampered the examination and evaluation of all tissues. However, histological examination of the adrenal gland, bladder, intestines, kidneys, liver, lungs, stomach and testes in the male long-finned pilot whale did not reveal any signs of neoplasia or inflammation. It was not possible to examine the lymph nodes or spleen due to severe autolysis. Parasites were also microscopically identified in the fore-stomach (*Pholeter gastrophilus)* and intestine (*Bolbosoma capitatum*). There were no signs of neoplasia or inflammation in the lungs of the female long-finned pilot whale, which was in an even more advanced state of decomposition, making most organs uninterpretable (kidneys, intestine, all lymph nodes, spleen, bladder). In the second stomach, there was a mild inflammatory infiltrate associated with the parasite remains but this was not severe enough to have caused death.

### Additional tests

No evidence for avian influenza virus or *Brucella ceti* infection was detected in either animal. The intestinal tract of the female long-finned pilot whale did not contain ESBL-producing *E*. *coli*.

### Diet analysis

In the GIT of the male long-finned pilot whale, six prey species were identified (in addition to the common sole found in the nasal cavity): European plaice (*Pleuronectes platessa)*, Atlantic herring (*Clupea harengus)*, common cuttlefish, river lampern (*Lampetra fluviatilis)*, sand goby (*Pomatoschistus minutus)* and brown shrimp.

The GIT of the female long-finned pilot whale was empty, except for three beaks of European squid (*Loligo vulgaris)* in the fore-stomach, indicating that this whale had not eaten for some time before ingesting the two soles found in the oesophagus and nasal cavity. No macro-plastic material or other foreign objects were visible in the GIT of either whale.

A complete overview of all prey items, the location of the prey species found, estimated sizes and masses are shown in Table 1.

## Discussion

Two rare strandings of long-finned pilot whales occurred within one month of each other on the Dutch coast and the necropsies revealed the same, remarkable cause of death: asphyxiation. Asphyxiation in cetaceans has been described for other smaller odontocetes, and we found only a single record of this cause of death in literature for *Globicephala* species: a 15 feet long (5 m) whale, considered by some to be a pilot whale, that stranded at Scheveningen, The Netherlands, in 1581, had “suffocated on a salmon” [24,25].

Six weeks prior to the first stranding described here, a pod of long-finned pilot whales, some 20–40 strong was sighted in the southern North Sea. The pod was first reported on the 10^th^ of November 2014 at Cley, Norfolk (UK), while three days later, a pod of long-finned pilot whales was observed in shallow waters off the coast of Blankenberge, Belgium, where they remained during the afternoon, and came within a couple of hundred meters from the shore. The behaviour of the animals (e.g. slow swimming and spy-hopping) and the similar group composition (including at least three calves and three large males) suggested that this was likely to have been the same group. Several sightings off the English east coast followed. On the 16^th^ of November, the pod was reported within the Thames estuary, near the Isle of Grain (Medway) and Sheppey Island. On the 18^th^ of November the group was observed in shallow waters north of the Thames within the Blackwater estuary in Essex. Using small vessels, UK volunteer rescue groups tried to prevent a mass stranding of the animals and the whales moved back into deeper waters. The pod was last observed off Essex on the 20^th^ of November. That same day, a juvenile female (2.18 m total length) long-finned pilot whale was found dead stranded at Goldhanger in the river Blackwater, Essex. The necropsy revealed that it was extremely emaciated and had a possible meningoencephalitis (inflammation of the brain and meninges) (UK strandings database, 2014). Pilot whales are known to be highly social animals, which remain in attendance of sick and injured group members [26]. We can only speculate about the reasons as to why this group entered the southern North Sea and stayed there for a considerable amount of time, but the compromised health of one individual could have played a role. With very few sizeable cephalopods available as prey in the southern North Sea [27], the whales may have had to resort to other, less usual prey species.

**Fig 3:**
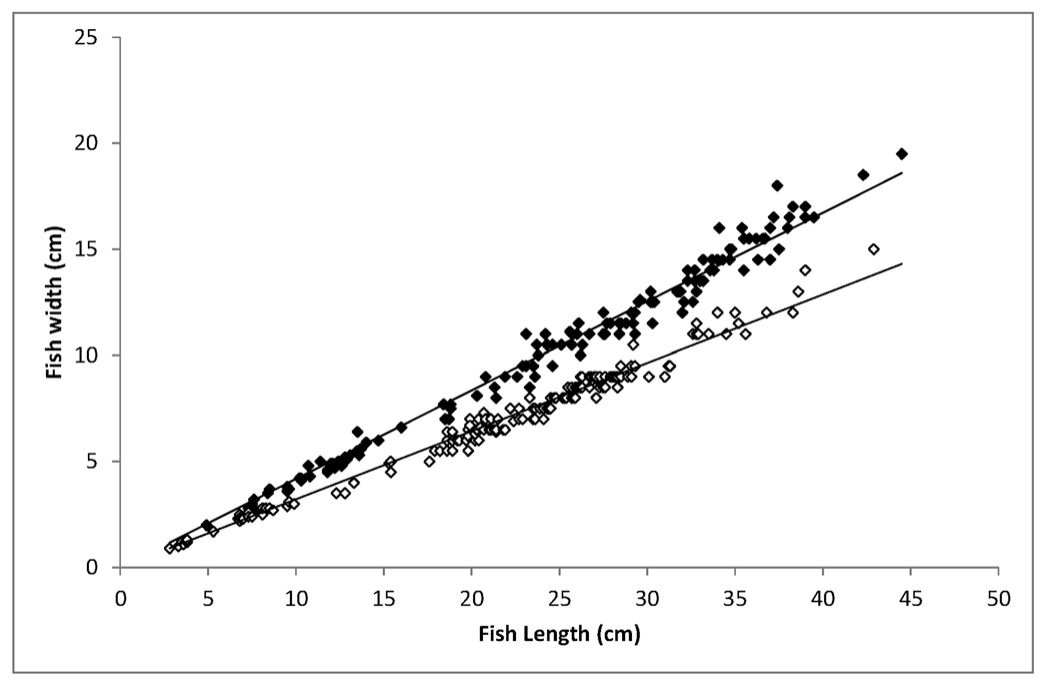
Body width as a function of total length in plaice and sole. Maximum body width (BW, cm) as a function of total length (TL, cm) in plaice (filled symbols: BW = 0.4178•TL, n = 138, R2 = 0.9819) and common sole (open symbols: BW = 0.3214•TL, n = 166138, R2 = 0.9813).

All mammals face the problem that their airway and digestive tracts intersect. In cetaceans which must swallow their prey under water, the airway runs centrally through the oesophagus, and prey items must pass the larynx via lateral food channels. However, large prey may be ingested through dislocation of the larynx from the nasopharynx [28,29]. This is a risky adaptation, as water may enter the airway and food may enter the nasal cavity, the latter possibly leading to fatal asphyxiation, as seen in the two cases described here. Flatfishes have wider bodies than most round fishes of similar biomass and ingesting even relatively small flatfish may require larynx dislocation. Common soles, however, are relatively narrow-bodied flatfish and should, at first sight, be easier to swallow than e.g. plaice, which were found in the male long-finned pilot whale (Table 1). Given that the plaice remains were found in the stomach, these fish apparently passed the larynx safely in this individual. The plaice that had been ingested by the male long-finned pilot whale must have had wider bodies than the common sole that caused asphyxiation in the larger female (Fig 3). This suggests that not the size, but the species of flatfish poses a particular risk during ingestion.

The common sole has been reported as prey only once in a long-finned pilot whale, in Normandy, France [12]. This sole had an estimated length of 40 cm and had apparently reached the stomach without problems. Common soles, however, are very agile fish, that may roll up in any direction when handled (*pers*. *obs*.) and that may jump up from the sea floor, head first, and down again in an “omega” shaped fashion [30]. Common soles of similar sizes to those found in our two cases are rather frequently predated on by harbour porpoises (*Phocoena phocoena*) in Germany. Here, six cases of fatal asphyxiation (among 133 fully necropsied porpoises) have been reported, including cases in which the soles reportedly entered the nasal cavity of the porpoise. In addition, eight unidentified flatfish were reported from asphyxiated porpoises that were not examined in full [31–33]. However, similar cases have not been reported in the UK (>2000 necropsies on harbour porpoises), The Netherlands (>1500 necropsies on harbour porpoises), and Belgium (>700 necropsies on harbour porpoises).

Several other odontocetes have been found with fish (other than soles) lodged in either their throat or oesophagus. These fish were often spiny, and had anchored themselves into the oesophageal wall, or were simply too large to be swallowed [34–40]. In such cases, the obstructing fish may dislodge or compress the larynx, resulting in asphyxia. However, a different process is likely to underlie the lodging of fish in the nasal cavity. The ingestion of common soles thus appears to be associated with risks that may reflect attempts of the fish to escape swallowing during which it somehow is able to enter an opening, i.e. the nasal cavity. Such behaviour is only to be expected from fishes that have a very flexible body. A consultation among colleagues working in post-mortem research on cetaceans yielded three possible additional cases: one flatfish and two American eels (*Anguilla rostrata*) in bottlenose dolphins (*Tursiops truncatus*) (Megan Stolen *in litt*., [39]). Eels also have very supple bodies and such prey may even, on occasion, successfully escape by exiting through the blowhole; clearly such cases will never come to light. Moreover, the predator may also successfully swallow such prey, as illustrated by the much larger common sole found in a long-finned pilot whale, in Normandy, France [12], or by the remains of river lamperns found in this study.

We consider that the soles involved in our cases may have actively entered into the nasal cavity during their efforts to avoid ingestion. Alternatively, the fish could have been pushed into the nasal cavity by the whale, through disarticulation, then re-articulation of the larynx into the blowhole [28]. This may also happen in response to an initial blocking of its airway. Forcibly exhaling in reaction to such a blockage is well-known in man, when food enters the trachea. However, with the larynx dislodged, the connection with the lungs is lost and the whale would be unable to “cough”, or “sneeze” to rid itself from such a blockage. Therefore, the soles must have either actively moved into the nasal cavity, or been pushed in by the larynx. It seems likely that the soles would move head-first towards the blowhole as moving backwards is prevented in this species by rough scales. However, in the second case, the fish was found with its tail protruding from the blowhole. This leaves open only two options: 1) the sole turned in the nasal cavity and got stuck with its tail pointing upwards, e.g. by getting stuck with its head into one of the lateral sacs adjacent to the nasal cavity, or 2) by postmortem entrance. The latter seems as implausible as the possibility of a sole moving into the blowhole from the oral cavity. Whilst scavenging fish, such as eels (*Anguilla sp*.) or Atlantic hagfish (*Myxine glutinosa*), might enter a carcass through any opening, soles are unlikely to do this as they are essentially small-mouthed worm-eaters [41].

To conclude, after ingesting the soles, the whales died as a result of asphyxiation following the soles attempt to escape: a lose-lose situation.
